# The effect of lumbar multifidus muscle degeneration on upper lumbar disc herniation

**DOI:** 10.3389/fsurg.2024.1323939

**Published:** 2024-11-12

**Authors:** Bingwen Wang, Lifei Xu, Peng Teng, Lin Nie, Hongwei Yue

**Affiliations:** ^1^ Department of Orthopedics, National Regional Medical Center, Dezhou People's Hospital, Qilu Hospital of Shandong University Dezhou Hospital, Dezhou, China; ^2^ Department of Clinical Laboratory, Lianyungang Maternal and Child Health Hospital, Affiliated Hospital of Kangda College of Southern Medical University, Lianyungang, China; ^3^ Department of Orthopedics, Chongming Hospital Affiliated to Shanghai University of Medicine and Health Sciences, Shanghai, China; ^4^ Department of Orthopedics, National Regional Medical Center, Qilu Hospital of Shandong University, Jinan, China

**Keywords:** upper lumbar disc herniation, lumbar multifidus muscle, lumbar multifidus muscle degeneration, fat infiltration, psoas major muscle

## Abstract

**Purpose:**

This study aimed to investigate the effect of lumbar multifidus muscle (MF) degeneration on upper lumbar disc herniation (ULDH).

**Methods:**

This study used 3.0T magnetic resonance imaging (MRI) T2 axial weighted images to retrospectively analyze 93 ULDH patients and 111 healthy participants. Sixty-five pairs of participants were included in this study using propensity score matching (PSM). Cross-sectional area, fat infiltration area, anteroposterior diameter (APD), lateral diameter (LD), cross-sectional area of the bilateral multifidus muscles at the corresponding level, intervertebral disc area at the corresponding section, and visual analog scale (VAS) score for low back pain (LBP). For inter-group comparisons, we used the *t*-test, analysis of variance (ANOVA), Mann–Whitney *U* test, Kruskal–Wallis test, chi-square test, or Fisher's exact test, according to the type of data. We used Pearson correlation analysis to study the correlation between the VAS score and related indicators, and established a predictive model for upper lumbar disc herniation using the receive operative characteristic (ROC) curve analysis method. Finally, univariate and multivariate logistic regression analyses were performed to establish a predictive model for the risk of high lumbar disc herniation.

**Results:**

We compared the fat areas at the lumbar vertebral levels L1/2, L2/3, and L3/4, as well as the left lateral diameter (LD) (MF), L1/2 left lumbar multifidus muscle index (LMFI), and L1/2 total fat infiltration cross-section area (TFCSA), and found significant differences between the case and control groups (*P* < 0.001). Furthermore, we observed a significant positive correlation (*P* < 0.05) between the VAS scores and multiple muscle indicators. Additionally, we developed ROC prediction models to assess the risk of lumbar intervertebral disc protrusion at the L1/2, L2/3, and L3/4 levels, with the results identifying L1/2 TFCSA, L2/3 TFCSA, and L3/4 relative psoas major muscle cross-section area (rPMCSA) as the most predictive indicators. Finally, univariate and multivariate logistic regression analyses showed that the L1/2 rPMCSA, L2/3 TFCSA were significantly associated with the risk of lumbar intervertebral disc protrusion in both models.

**Conclusion:**

Degeneration of the MF is significantly correlated with the occurrence of ULDH, and the larger the area of fat infiltration in the MF, the more obvious the lower back pain in ULDH patients. In addition, TFCSA can serve as an indicator of the occurrence of ULDH.

## 1 Introduction

Lumbar disc herniation (LDH) is a highly prevalent degenerative spinal disease. The protruding nucleus pulposus compresses or stimulates the sinus nerve and nerve roots, causing clinical symptoms, such as LBP, lower limb pain, and numbness. It is the primary cause of low back pain and sciatica in humans (1). With changes in work and lifestyle habits, the proportion of patients with LDH has sharply increased, with patients now tending to be younger, damaging their physical and mental health, and has become one of the main diseases threatening human health (2, 3).

More than 90% of patients have LDH at the L4/5 or L5/S1 levels, which we refer to as lower LDH (LLDH). Less than 10% of the remaining LDH occur in the L3/4 or above, collectively known as upper LDH (ULDH) (4). LLDH is usually caused by gender, weight, occupation, and lifestyle habits, with intervertebral disc degeneration and chronic strain being the primary cause (5). The exact pathogenesis of ULDH remains unclear, and there is relatively little research on this topic. Some spinal biomechanical studies have shown that the main cause of LDH caused by lumbar disc degeneration is lumbar spinal imbalance. This is closely related to the MF, which controls spinal curvature and maintains mechanical stability (6).

The lumbar MF is the largest posterior muscle group in the lumbosacral region, which is extremely important for providing lumbar segment stability and is a dynamic stabilizer of the lumbar spine (7). The MF strengthens lumbar lordosis and resists lumbar flexion during lumbar spine rotation, thereby regulating the distribution of the intervertebral load and pressure. Many studies have explored the impact of paraspinal muscles on lumbar degenerative diseases (8, 9). The degeneration of the MF can lead to changes in the original biomechanical relationship, increasing the load on the intervertebral disc, and ultimately leading to LDH (10). Studies have shown that patients observe a higher rate of fat infiltration into paravertebral muscles. The paraspinal muscles of patients with LDH contain more stem cells that have higher levels of fibroblasts and adipogenic gene expression (11). Degeneration and fat infiltration in the paravertebral muscles can exacerbate symptoms of LBP. Paravertebral muscle fat infiltration is associated with small-joint degeneration and vertebral space stenosis. Patients with higher degrees of lumbar facet joint degeneration showed significantly increased infiltration of paravertebral fat. Related studies have shown that polyfiber muscle fat infiltration is an independent factor that influences the degree of intervertebral disc degeneration (12). Shi al. showed a strong positive correlation between the Pfirmann classification and MF fat infiltration (Rho = 0.57, *P* < 0.001), with a moderate positive correlation with the psoas major muscle (PM) (Rho = 0.31, *P* < 0.001) (13). In addition, muscle symmetry has become a focus of attention (11). Some studies have suggested that cross-sectional area (CSA) asymmetry of the MF can be used to indicate potential spinal abnormalities (14, 15). Relevant studies have explored the correlation between the PM muscle and lumbar spine imbalance. It is generally believed that the PM maintains lumbar spine stability. The bilateral asymmetry or atrophy of PM can also lead to uneven force distribution in the lumbar stability system, increasing the shear stress of the intervertebral disc, and ultimately leading to disc herniation (16, 17).

With the emergence of high-resolution lumbar computed tomography (CT) and magnetic resonance imaging (MRI), we have gained a deeper understanding of the pathogenesis of LDH. In particular, there is a close correlation between lumbar degeneration and the related supporting structures. MRI has been effectively able to distinguish between muscle and adipose tissue through threshold segmentation technology, and the effectiveness and reliability of indirect evaluation of the characteristics of the lumbar MF have been shown in relevant studies (10). Studying the relationship between MF degeneration and spinal-related structures may provide significant guidance for the prevention or treatment of spinal lesions. In the past, most studies on MF and LDH focused mainly on the lower lumbar spine segment, with only a few studies analyzing the relationship between MF degeneration and ULDH. The parameters were limited, and differences in the measurement results were not comparable (18–20). In some of these studies, Fuji software was used for image analysis (21). This study aimed to analyze the impact of MF degeneration on ULDH by comparing the MR features of MF between patients with ULDH and healthy individuals. This comparison contributes to understanding how MF degeneration facilitates the development or progression of ULDH, filling a gap in related research fields.

## 2 Methods

### 2.1 Participants

This cross-sectional comparative study was conducted at the Qilu Hospital of Shandong University, Dezhou Hospital. All research procedures were conducted in accordance with the principles of the Declaration of Helsinki and approved by the Qilu Hospital of Shandong University Dezhou Hospital Ethics Committee.

This study included 204 patients treated for LDH at the Qilu Hospital of Shandong University Dezhou Hospital from January 2021 to June 2023, as well as healthy participants undergoing physical examination. The inclusion criteria for the ULDH group were as follows: (1) age 20–75 years, (2) lumbar spine MRI examination; and (3) LDH at L1/2, L2/3, and L3/4. The purpose of this study was to explore the correlation between ULDH patients and MF degeneration, and there were no other factors included if they met any of the following criteria: (1) MRI showed lumbar spondylolisthesis (>3 mm), (2) spinal column deformity (for example, scoliosis >10°), (3) spinal fracture, (4) history of lumbar surgery or lumbar back treatment (epidural injection, traditional Chinese medicine treatment), (5) spinal tuberculosis, (6) history of tumors, (7) history of rheumatism (rheumatoid arthritis, ankylosing spondylitis), (8) history of infection, (9) cardiovascular, pulmonary, cerebrovascular, and neuromuscular disorders, and (10) incomplete imaging data or poor image quality. The control group included healthy subjects who underwent MR examination at the outpatient department or physical examination centre of the hospital where the study was conducted. The inclusion criteria for the control group were as follows: (1) Age 20–75 years, (2) lumbar spine MR examination, and (3) no history of lumbar degenerative diseases such as LDH. The exclusion criteria were as follows: (1) medical history of spinal surgery; (2) infection, tumor, spinal trauma, spinal tuberculosis, etc.; and (3) unclear MR images. All participants’ medical records included complete medical history, physical examination data (including age, sex, body height, and body weight), and lumbar spine MRI findings. All the participants provided written informed consent.

### 2.2 Data measurements

**Figure 1:**
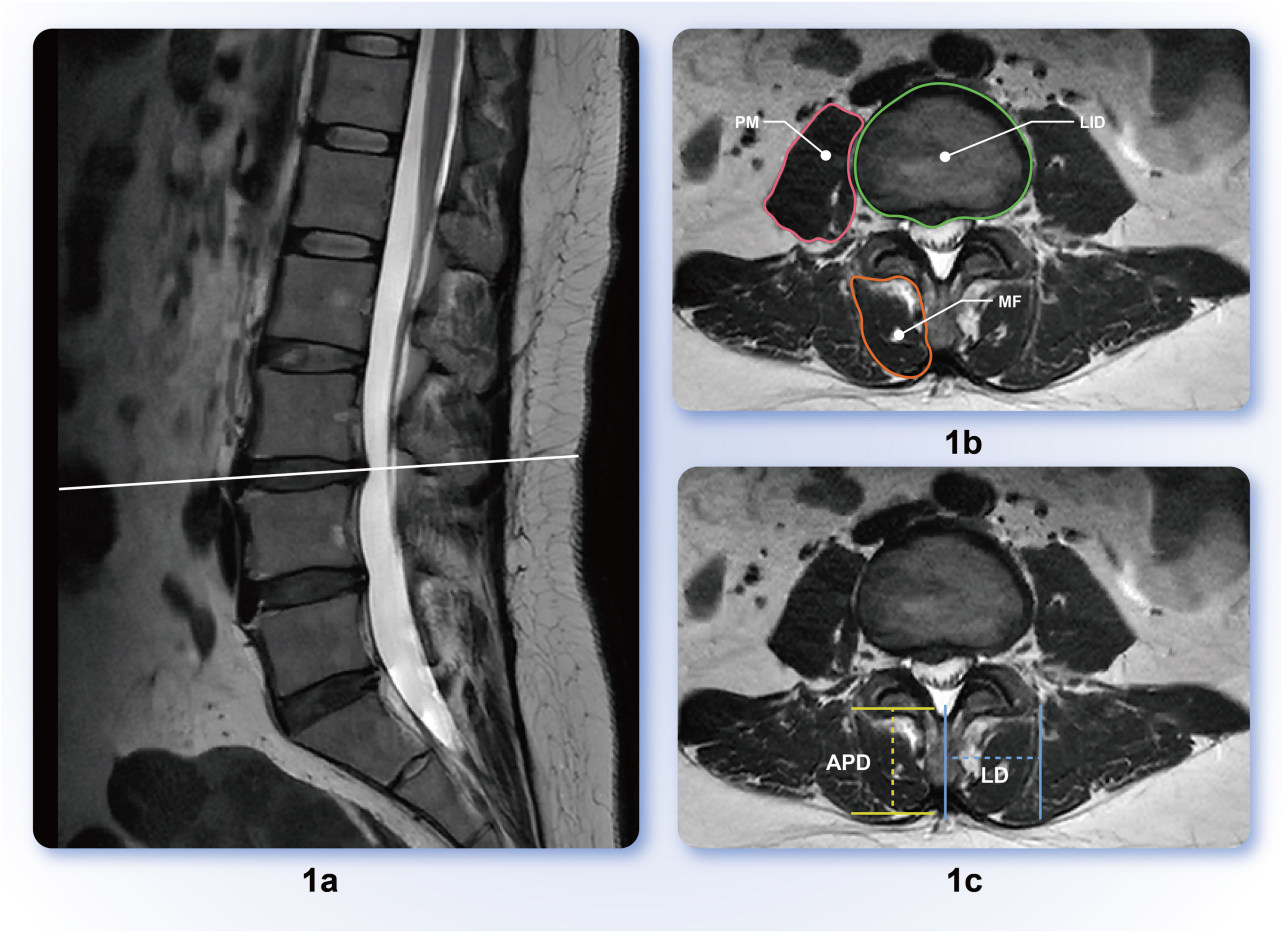
**(a)** Sagittal representation of intervertebral disc protrusion segment; **(b)** lumbar Multifidus muscle (MF), psoas major muscle (PM), lumbar intervertebral disc (LID) delineation scope diagram; **(c)** anteroposterior diameter (APD), lateral diameter (LD) measurement schematic; APD is the vertical distance between the two parallel lines before and after MF, and LD is the horizontal distance from the most prominent point on the outer edge of the multifidus muscle to the midline of the spinous process.

The MRI data of all the participants reviewed in this study were collected using a 3.0T MRI scanner (uMR780, Shanghai Lianying, China), with the scanning positions in the supine position when the lumbar spine was neutral. Measure axial T2 weighted slice images passing through the center level of the L1/2, L2/3, and L3/4 intervertebral discs were obtained using Fuji software. The target parameters were the bilateral MF CSA, fat infiltration area, antioxidant diameter, lateral diameter, bilateral PM CSA, corresponding lumbar intervertebral disc (LID) area, and lumbago visual analog scale (VAS) (Figure 1). The corresponding parameters of the control group in the same plane were measured using a data-matching method.

To reduce the impact of body size, sex, and other factors on measured anatomical parameters, this study also included relative fat CSA (rFCSA), which is the ratio of fat CSA to the corresponding cross-sectional intervertebral disc area; the relative PM CSA [rCSA (PM)] is the ratio of the CSA of the PM to the corresponding CSA of the intervertebral disc.

### 2.3 Statistical analysis

For data analysis, we used IBM SPSS software (version 27.0) and R software version 4.2.2. For continuous data conforming to a normal distribution, descriptive statistics were represented as the mean ± standard deviation (*x¯* ± SD). Inter-group comparisons were conducted using the *t*-test for two groups and analysis of variance (ANOVA) for multiple groups. In instances where the data displayed a non-normal distribution, summary statistics were articulated using the median (interquartile range) [*M*(*Q*)]. Inter-group comparisons for non-normally distributed data were performed using non-parametric tests, specifically the Mann–Whitney *U* test for two groups and the Kruskal–Wallis test for multiple groups. For count data, frequencies (percentages) [*n* (%)] were used for data representation, and inter-group comparisons were performed using either the chi-square test or Fisher's exact test, depending on the sample size and data characteristics.

Furthermore, Pearson correlation analysis was used to investigate the correlation between VAS scores and relevant indicators. We constructed a predictive model for lumbar disc herniation using ROC curve analysis. The performance of predictors was assessed using the ROC area under the curve (AUC), sensitivity, specificity, and Youden's index values. Additionally, we established predictive models for high-level lumbar disc protrusion risk using univariate and multivariate logistic regression analyses.

Propensity scores were calculated using logistic regression, considering the aforementioned demographic and clinical characteristics. We performed 1:1 greedy nearest neighbor matching with a caliper of 0.1. This method functionally relies on the R package MatchIt. A distance was computed between unit of one group and another, and, one by one, each unit was assigned a control unit as a match. The matching was “greedy” in the sense that no action was taken to optimize an overall criterion; each match was selected without considering the other matches that might occur subsequently. After matching, we performed a comparison of clinical outcomes between the two groups using appropriate statistical tests.

## 3 Results

Demographic and clinical characteristics of the patients, such as age, body height, body weight, body mass index (BMI), and sex, were collected for both groups. To reduce the impact of potential confounding factors, we matched 65 pairs of ULDH patients and healthy participants using propensity score matching (PSM). This statistical technique aimed to balance the covariates between the treatment groups, ensuring that any observed differences in the outcomes were more likely to be attributed to the study itself rather than to the influence of confounding factors.

Table 1 shows the baseline characteristics of the study population after PSM screening. Sex distribution was comparable between the control and case groups, with 57% and 51% females, respectively. Similarly, there was no significant difference in age between the groups (median = 58.0 years). Body height, weight, BMI, and various muscle measurements including total area, muscle area, fat area, length, and width were similar between the groups. Additionally, significant differences were found in the fat areas at the L1/2 right/left, L2/3 right/left, and L3/4 right/left levels, indicating a higher fat area in the case group (*P* < 0.001, *P* = 0.002, *P* < 0.001, and *P* = 0.007, respectively). Statistically significant differences were observed between the L2/3 Left LD (MF) groups. The disc areas and VAS scores also showed statistically significant differences between the groups. These findings suggest that sex, age, and body measurements were similar between the groups, but that there were distinct differences in protrusion direction and fat distribution at various lumbar levels, which may have clinical implications.

**Table 1:** Participants demographics and baseline characteristics.

Characteristic	Group		*P*-value^b^
	Control group, *N* = 65^a^	Study group, *N* = 65^a^	
Gender			0.482
Female	37 (57%)	33 (51%)	
Male	28 (43%)	32 (49%)	
Age	58.0 (55.00, 63.00)	58.0 (54.00, 63.00)	0.885
Height	1.6 (1.57, 1.68)	1.6 (1.58, 1.67)	0.841
Weight	60.7 ± 5.99	61.2 ± 5.73	0.629
BMI	23.1 ± 2.27	23.2 ± 2.13	0.801
Protruding direction			
L	—	36 (55%)	
R	—	29 (45%)	
TCSA (MF)			
L1/2R	2.3 (1.95, 2.71)	2.3 (1.79, 2.63)	0.944
L1/2l	2.4 (2.00, 2.66)	2.4 (1.92, 2.72)	0.923
L2/3R	3.4 (2.89, 3.95)	3.4 (2.74, 3.86)	0.746
L2/3l	3.4 (2.91, 3.98)	3.4 (2.60, 4.02)	0.620
L3/4R	5.7 (4.98, 6.48)	5.6 (5.09, 6.00)	0.878
L3/4l	5.5 (4.89, 6.35)	5.5 (4.45, 6.53)	0.710
FCSA (MF)			
L1/2R	0.3 (0.16, 0.42)	0.5 (0.40, 0.67)	<0.001*
L1/2l	0.3 (0.15, 0.44)	0.5 (0.41, 0.73)	<0.001*
L2/3R	0.4 (0.23, 0.58)	0.6 (0.41, 0.83)	<0.001*
L2/3l	0.4 (0.25, 0.57)	0.7 (0.46, 0.74)	0.002*
L3/4R	0.8 (0.49, 1.16)	1.2 (0.72, 1.54)	0.047*
L3/4l	0.8 (0.45, 1.11)	1.2 (0.92, 1.32)	0.007*
APD (MF)			
L1/2R	2.2 ± 0.29	2.1 (0.38	0.284
L1/2l	2.1 (2.01, 2.40)	2.0 (1.87, 2.32)	0.083
L2/3R	2.6 ± 0.35	2.7 ± 0.47	0.399
L2/3l	2.6 ± 0.35	2.6 ± 0.55	0.669
L3/4R	3.3 ± 0.52	3.3 ± 0.40	0.940
L3/4l	3.3 ± 0.53	3.2 ± 0.37	0.700
LD (MF)			
L1/2R	1.9 (1.82, 2.06)	1.9 (1.77, 2.02)	0.892
L1/2l	1.9 (1.81, 2.08)	2.0 (1.79, 2.06)	0.550
L2/3R	2.1 (1.96, 2.21)	2.2 (1.99, 2.33)	0.215
L2/3l	2.1 ± 0.23	2.2 ± 0.25	0.043*
L3/4R	2.5 ± 0.28	2.6 ± 0.31	0.227
L3/4l	2.5 ± 0.28	2.6 ± 0.34	0.391
CSA (PM)			
L1/2	4.9 (3.99, 6.70)	5.6 (3.90, 8.25)	0.461
L2/3	10.8 (8.83, 14.36)	11.8 (10.84, 14.37)	0.159
L3/4	16.8 (13.83, 22.80)	15.4 (12.42, 20.68)	0.400
CSA (LID)			
L1/2	13.8 (12.46, 14.96)	18.2 (15.89, 19.59)	<0.001*
L2/3	15.2 ± 2.22	20.7 ± 2.41	<0.001*
L3/4	16.0 (14.30, 17.84)	18.6 (16.17, 20.18)	0.010*
VAS	2.0 (1.00, 3.00)	5.0 (3.00, 6.00)	<0.001*

^a^ *n* (%).

^b^ Pearson's Chi-squared test; Wilcoxon rank sum test; Welch Two Sample *t*-test.

* *P* < 0.05.

BMI, body mass index; TCSA, total cross-sectional area; R, right; L, left; FCSA, fat infiltration cross-sectional area; APD, anteroposterior diameter; LD, lateral diameter; LID, lumbar intervertebral disc.

Table 2 presents the baseline characteristics of the control and case groups. L1/2 right lumbar multifidus muscle index (RMFI) was not significantly different between the two groups (*P* = 0.121). The median L1/2 RMFI was similar in both groups at 0.9 [interquartile range (IQR): 0.80–0.95] for the control group and 0.9 (IQR: 0.79–1.03) for the case group. In contrast, the L1/2 lumbar multifidus muscle index (MFI) demonstrated a significant difference between the two groups (*P* = 0.034). The median L1/2 MFI was higher in the case group (1.0, IQR: 0.88–1.06) compared to that in the control group (0.9, IQR: 0.82–0.97). L1/2 TFCSA also showed a significant difference (*P* < 0.001), with higher median values observed in the case group (1.1, IQR: 0.87–1.39) compared to the control group (0.6, IQR: 0.30–0.91). Other characteristics such as L1/2 relative total fat infiltration cross-section area (rTFCSA), L1/2 psoas major muscle cross-section area (PMCSA), L1/2 rPMCSA, L1/2 relative right fat infiltration cross-section area (rRFCSA), and L1/2 relative right fat infiltration cross-section area (rLFCSA) did not show significant differences between the groups. Similar patterns were observed for the characteristics at the L2/3 and L3/4 levels.

**Table 2:** Participants demographics and baseline characteristics.

Characteristic	Group		*P*-value^a^
	Control group, *N* = 65	Study group, *N* = 65	
MFI			
L1/2R	0.9 (0.80, 0.95)	0.9 (0.79, 1.03)	0.121
L1/2l	0.9 (0.82, 0.97)	1.0 (0.88, 1.06)	0.034*
L2/3R	0.8 (0.74, 0.91)	0.8 (0.73, 0.95)	0.787
L2/3l	0.8 (0.74, 0.88)	0.8 (0.77, 0.99)	0.431
L3/4R	0.8 (0.70, 0.84)	0.8 (0.73, 0.86)	0.292
L3/4l	0.7 (0.70, 0.82)	0.8 (0.74, 0.84)	0.180
TFCSA (MF)			
L1/2	0.6 (0.30, 0.91)	1.1 (0.87, 1.39)	<0.001*
L2/3	0.7 (0.50, 1.17)	1.3 (0.83, 1.68)	<0.001*
L3/4	1.4 (0.91, 2.41)	2.5 (1.89, 2.93)	0.009*
rFCSA (MF)			
L1/2T	0.0 (0.02, 0.07)	0.1 (0.05, 0.07)	0.009*
L1/2R	0.0 (0.01, 0.03)	0.0 (0.02, 0.04)	0.008*
L1/2l	0.0 (0.01, 0.04)	0.0 (0.02, 0.04)	0.021*
L2/3T	0.0 (0.03, 0.08)	0.1 (0.04, 0.08)	0.196
L2/3R	0.0 (0.02, 0.04)	0.0 (0.02, 0.04)	0.107
L2/3l	0.0 (0.02, 0.04)	0.0 (0.02, 0.04)	0.358
L3/4T	0.1 (0.06, 0.15)	0.1 (0.11, 0.16)	0.138
L3/4R	0.0 (0.03, 0.07)	0.1 (0.04, 0.08)	0.301
L3/4l	0.0 (0.03, 0.07)	0.1 (0.06, 0.08)	0.099
rCSA (PM)			
L1/2	0.4 (0.32, 0.47)	0.3 (0.22, 0.44)	0.028*
L2/3	0.7 (0.59, 0.90)	0.6 (0.51, 0.69)	0.003*
L3/4	1.1 (0.88, 1.28)	0.8 (0.72, 0.93)	0.003*

^a^ Wilcoxon rank sum test; Welch Two Sample *t*-test.

* *P* < 0.05.

MFI, lumbar multifidus muscle index, LD/APD; RFCSA, right fat infiltration cross-section area; LFCSA, left fat infiltration cross-section area; TFCSA, total fat infiltration cross-sectional area; RFCSA + LFCSA; rFCSA, relative fat infiltration cross-section area, FCSA/LIDCSA; T, Total; PM, psoas major muscle; rCSA (PM), relative PM cross-section area, PMCSA/LIDCSA.

**Figure 2:**
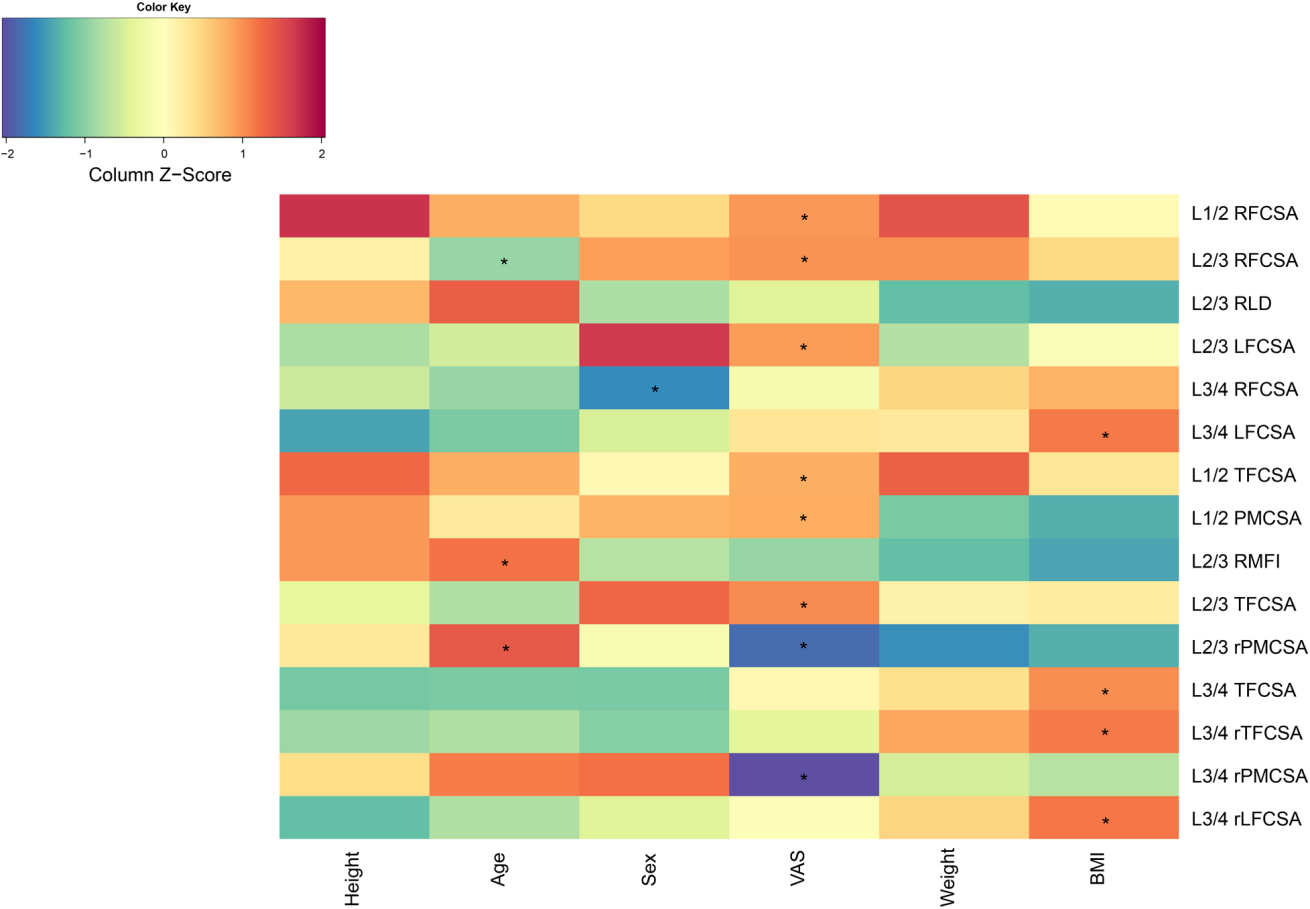
Correlation analysis chart. BMI, body mass index; LFCSA, left fat infiltration cross-sectional area; MF, multifidus muscle; PMCSA, psoas major muscle cross-sectional area; RFCSA, right fat infiltration cross-sectional area; RLD, right lateral diameter; RMFI, right lumbar multifidus muscle index; TFCSA, total fat infiltration cross-sectional area; VAS, visual analog score.

VAS scores displayed notable correlations with various spinal anatomical characteristics. Specifically, the L1/2 right fat infiltration cross-sectional area (RFCSA) and the L2/3 RFCSA were both significantly associated with VAS, yielding correlations of *r* = 0.294 (*P* < 0.01) and *r* = 0.299 (*P* < 0.01), respectively. The L2/3 left MF fat area also displayed a significant relationship with the VAS score (*r* = 0.290, *P* < 0.01). Other anatomical markers, including L1/2 TFCSA (*r* = 0.264,*P* < 0.05), L1/2 PMCSA (*r* = 0.268, *P* < 0.05), L2/3 TFCSA (*r* = 0.308,*P* < 0.01), L2/3 rPMCSA (*r* = 0.214,*P* < 0.05), and L3/4 rPMCSA (*r* = −0.252, *P* < 0.05) showed significant correlations with VAS (Figure 2).

**Figure 3:**
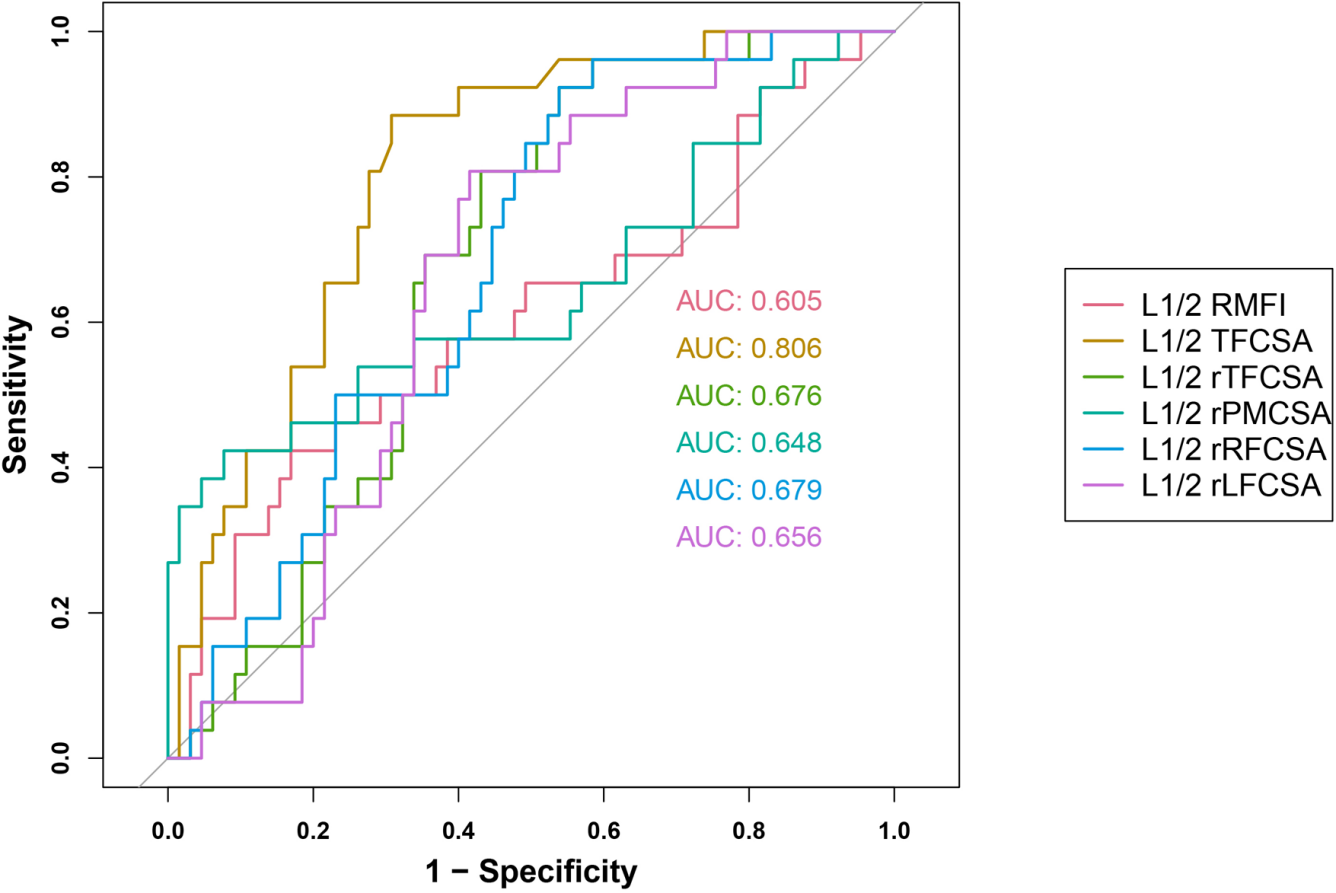
L1/2 receiver operating characteristic curve. AUC, area under the curve; rLFCSA, relative left fat infiltration cross-sectional area; rRFCSA, relative right fat infiltration cross-sectional area; LMFI, left lumbar multifidus muscle index; rPMCSA, relative psoas major muscle cross-sectional area; rTFCSA, relative total fat infiltration cross-sectional area.

We evaluated six predictor groups, including L1/2 LMFI, L1/2 TFCSA, L1/2 rTFCSA, L1/2 rPMCSA, L1/2 rRFCSA, and L1/2 rLFCSA (Figure 3).

In the L1/2 LMFI group, the AUC was 0.643 [95% confidence interval (CI): 0.506–0.781], with an identified cut-off value of 0.9550. This generated a sensitivity of 0.577, a specificity of 0.723, and a Youden's index of 0.300.

The L1/2 TFCSA group exhibited the highest AUC of 0.806 (95% CI: 0.714–0.898) and a cutoff value of 0.7500, accompanied by the highest sensitivity of 0.885, specificity of 0.692, and Youden's index of 0.577.

The L1/2 rTFCSA group had an AUC of 0.676 (95% CI: 0.566–0.785) at a cutoff value of 0.0450. The sensitivity and specificity were 0.808 and 0.554, respectively, and Youden's index was 0.362.

Furthermore, the L1/2 rPMCSA predictor demonstrated an AUC of 0.648 (95% CI: 0.506–0.790), with a cutoff value of 0.2550. Despite the lower sensitivity of 0.385, this group showed the highest specificity of 0.938, along with a Youden's index of 0.323.

For the L1/2 rRFCSA group, an AUC of 0.679 (95% CI: 0.567–0.790) was found, with a cutoff value of 0.0150, high sensitivity of 0.962, specificity of 0.400, and Youden's index of 0.362.

Finally, L1/2 rLFCSA was characterized by an AUC of 0.656 (95% CI: 0.544–0.769), a cutoff value of 0.0250, a sensitivity of 0.692, a specificity of 0.646, and a Youden's index of 0.338.

**Figure 4:**
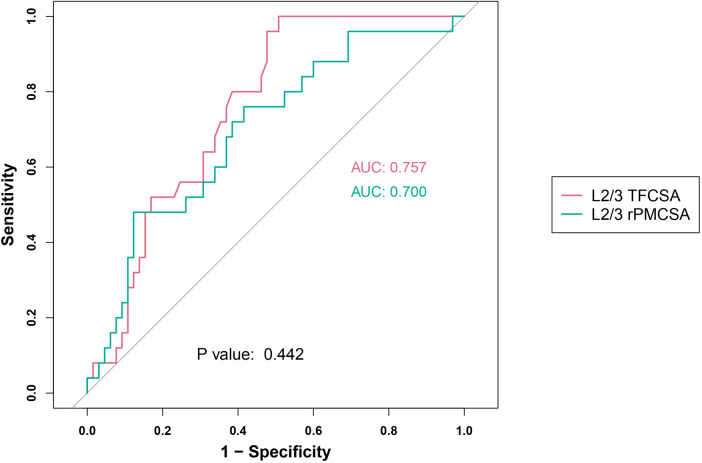
L2/3 receiver operating characteristic curve. AUC, area under the curve; TFCSA, total fat infiltration cross-sectional area; rPMCSA, relative psoas major muscle cross-sectional area.

We assessed two predictor groups: L2/3 TFCSA and L2/3 rPMCSA (Figure 4). The performance of predictors was evaluated using the ROC AUC, sensitivity, specificity, and Youden's index values.

For the L2/3 TFCSA group, we observed an AUC of 0.757 (95% CI: 0.659–0.855), with an identified cutoff value of 0.7050. The resulting sensitivity was high (0.960), and the specificity was 0.523. The Youden index for this group was 0.483.

For the L2/3 rPMCSA group, the AUC was 0.700 (95% CI: 0.580–0.821) at a cut-off value of 0.6950. This group showed a sensitivity of 0.760, a specificity of 0.585, and a Youden's index of 0.345.

**Figure 5:**
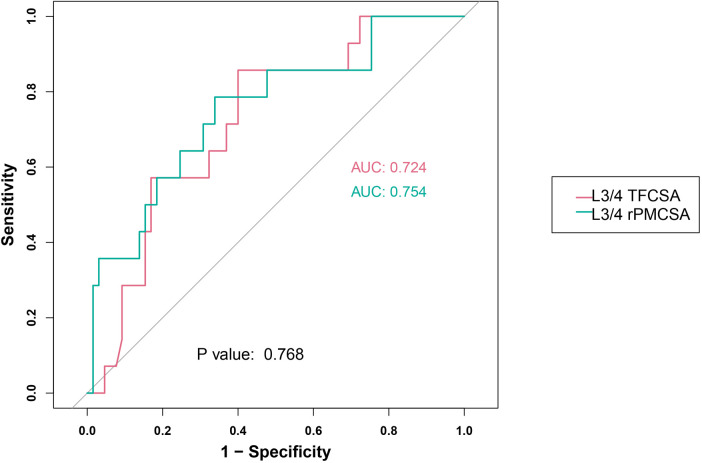
L3/4 receiver operating characteristic curve. AUC, area under the curve; TFCSA, total fat infiltration cross-sectional area; rPMCSA, relative psoas major muscle cross-sectional area.

Our research results incorporated the assessment of two predictor groups: L3/4 TFCSA and L3/4 rPMCSA (Figure 5). The performance indicators used to assess these groups were the ROC AUC, sensitivity, specificity, and Youden's index.

For the L3/4 TFCSA group, an AUC of 0.724 was noted (95% CI: 0.588–0.859). The optimum cutoff value was 1.8500, resulting in a sensitivity of 0.857 and a specificity of 0.600. The Youden index, was 0.457.

In the L3/4 rPMCSA group, the AUC was 0.754 (95% CI: 0.607–0.900), with an ideal cutoff value of 0.9350. The sensitivity and specificity in this group were 0.786 and 0.662, respectively. Youden's index for this predictor was 0.447.

These results suggest contrasting performances of the two examined predictors across various operational characteristics.

A range of characteristics was assessed using both univariate and multivariate approaches. In the total sample of 130 participants, age showed no significant relationship with the risk of ULDH in the univariate model [odds ratio (OR) = 1.00, 95% CI = 0.95, 1.06, *P* = 0.989]. However, upon multivariate adjustment for a reduced sample size of 91 participants, age demonstrated a modest yet significant association (O*R* = 1.11, 95% CI = 1.02, 1.20, *P* = 0.015).

In terms of sex, men appeared to have slightly higher odds of ULDH in both the univariate (O*R* = 1.28, 95% CI = 0.64, 2.57, *P* = 0.482) and multivariate (O*R* = 1.66, 95% CI = 0.61, 4.68, *P* = 0.324) models, although these values were not statistically significant.

**Figure 6:**
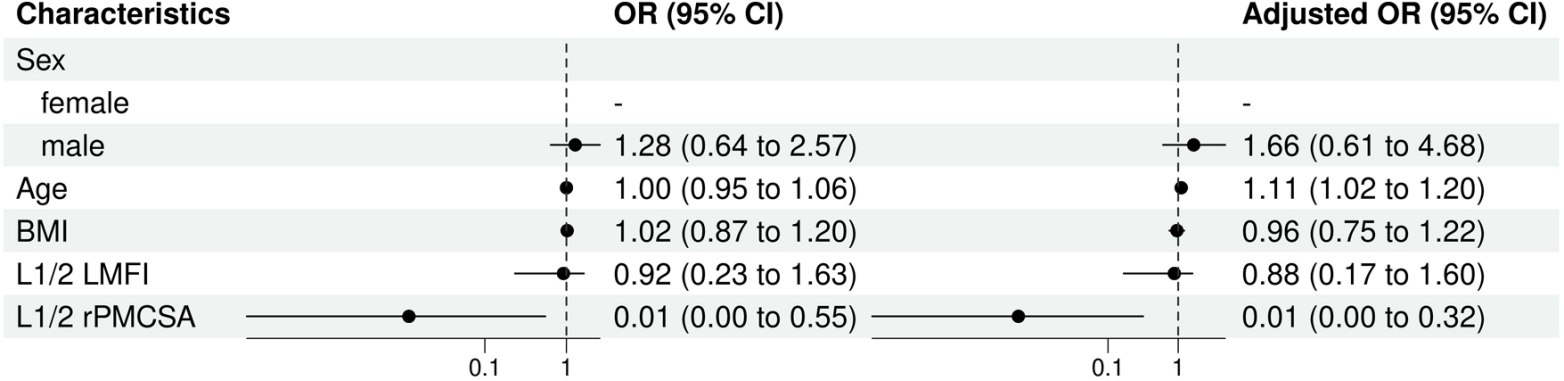
L1/2 univariate and multivariate analysis of influencing factors (logistic regression). BMI, body mass index; CI, confidence interval; LMFI, left lumbar multifidus muscle index; OR, odds ratio; rPMCSA, relative psoas major muscle cross-sectional area; PMCSA, psoas major muscle cross-sectional area.

BMI and L1/2 MFI measurements did not significantly contribute to the corresponding univariate or multivariate models. L1/2 rPMCSA, however, showed a substantial and highly significant negative association with the risk of ULDH in both models (O*R* = 0.01, *P* = 0.040 univariate; O*R* = 0.01, *P* = 0.020 multivariate) (Figure 6).

**Figure 7:**
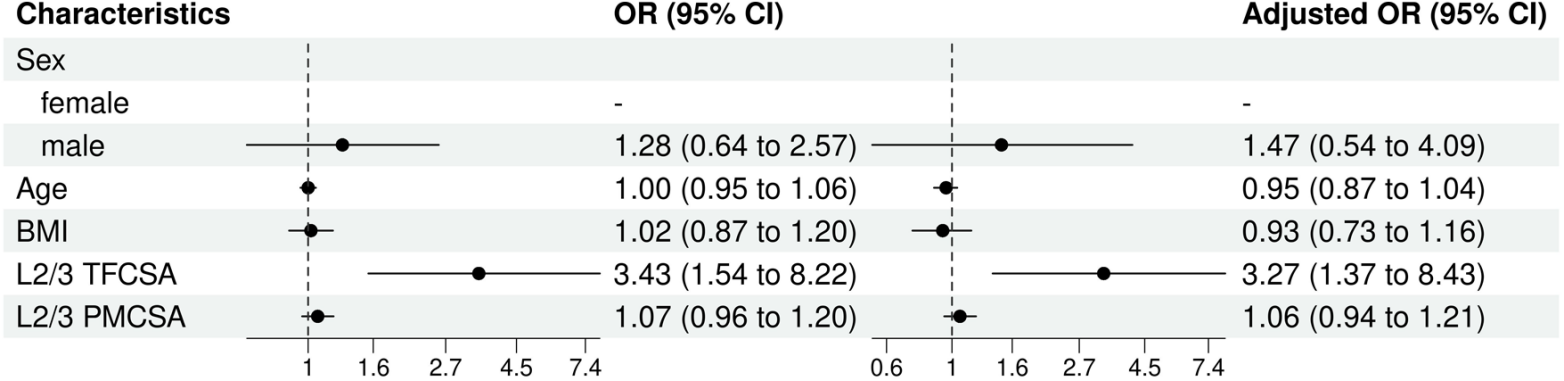
L2/3 univariate and multivariate analysis of influencing factors (logistic regression). BMI, body mass index; TFCSA, total fat infiltration cross-sectional area; CI, confidence interval; OR, odds ratio; PMCSA, psoas major muscle cross-sectional area.

Univariate and multivariate logistic regression analysis results indicated differences in the predictive factors contributing to the risk of ULDH, especially L2/3 TFCSA (Figure 7).

In both models, sex demonstrated comparable trends although the differences were not statistically significant. Males showed moderately higher odds of experiencing ULDH than females, but the difference was not statistically significant (univariable OR: 1.28, 95% CI: 0.64–2.57, *P* = 0.48; multivariable OR: 1.47, 95% CI: 0.54–4.09,*P* = 0.45).

As for age and BMI, neither presented a significant influence on ULDH in both models (age, univariable OR: 1.00, 95% CI: 0.95–1.06, *P* = 0.99; multivariable OR: 0.95, 95% CI: 0.87–1.04, *P* = 0.31; BMI, univariable OR: 1.02, 95% CI: 0.87–1.20, *P* = 0.80; multivariable OR: 0.93, 95% CI: 0.73–1.16, *P* = 0.53).

The most notable finding, was the statistically significant influence of the L2/3 TFCSA on ULDH occurrence in both models (univariable OR: 3.43, 95% CI: 1.54–8.22, *P* = 0.003; multivariable OR: 3.27, 95% CI: 1.37–8.43, *P* = 0.010). This indicated that an increase in L2/3 TFCSA was significantly associated with increased odds of ULDH.

In contrast, L2/3 PMCSA does not significantly influence ULDH in either model (univariable OR: 1.07, 95% CI: 0.96–1.20, *P* = 0.22; multivariable OR: 1.06, 95% CI: 0.94–1.21, *P* = 0.32).

In summary, among the factors assessed, only the L2/3 TFCSA was significantly associated with the risk of ULDH.

**Figure 8:**
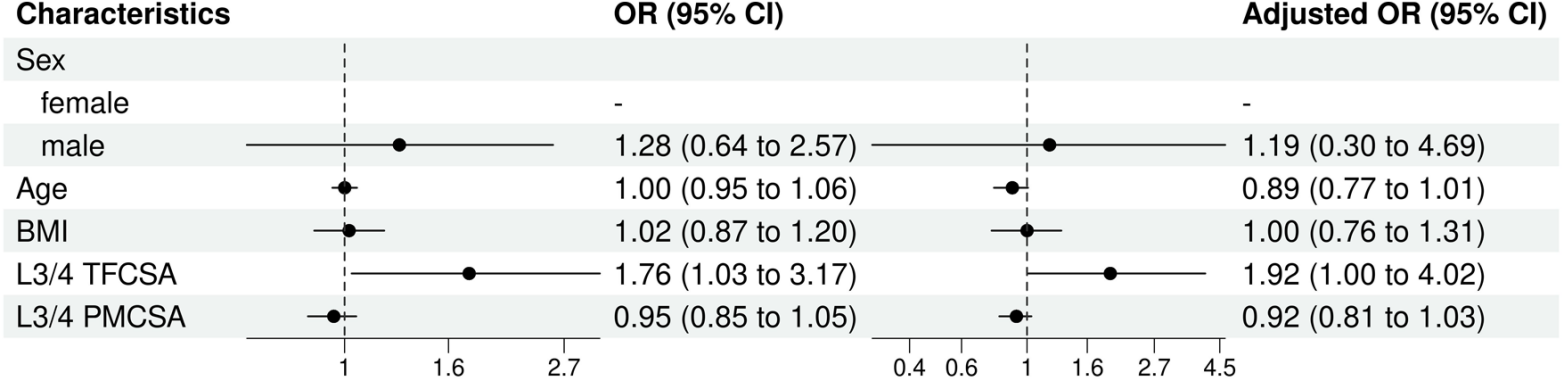
L3/4 univariate and multivariate analysis of influencing factors (logistic regression). BMI, body mass index; CI, confidence interval; TFCSA, total fat infiltration cross-sectional area; OR, odds ratio; PMCSA, psoas major muscle cross-sectional area.

Univariate and multivariate logistic regression analyses elucidated the different characteristics that influenced the risk of ULDH in the sample population (Figure 8).

For sex, although males presented slightly higher odds of experiencing ULDH than females, this difference was not statistically significant (OR 1.28, 95% CI: 0.64–2.57, *P* = 0.48). Age (OR: 1.00, 95% CI: 0.95–1.06, *P* = 0.99) or BMI (OR: 1.02, 95% CI: 0.87–1.20, *P* = 0.80) presented a statistically significant impact on ULDH occurrence in the univariable analysis.

In multivariate analysis, L3/4 TFCSA had a significant influence on the risk of ULDH. TFCSA (univariable OR: 1.76, 95% CI: 1.03–3.17). This influence was not statistically significant in the univariable analysis (OR:1.92, 95% CI: 1.00–4.02, *P* = 0.06).

L3/4 PMCSA, in both univariable and multivariable models, had no statistically significant effect on ULDH (univariable OR: 0.92, 95% CI: 0.81–1.03, *P* = 0.19; multivariable OR:OR: 0.95, 95% CI: 0.85–1.05,*P* = 0.37).

## 4 Discussion

PSM was used to explore the effect of MF degeneration on the risk of ULDH. Our results showed that compared to the healthy control group, ULDH patients had a significantly increased fat infiltration area in the responsible segment of the MF, and the larger the area of fat infiltration in the MF, the higher the VAS score for low back pain in ULDH patients.

This study further analyzed the impact of MF degeneration on ULDH. The ROC prediction model results showed that in the L1/2 and L2/3 groups, the AUC of the TFCSA were 0.806 (95% CI: 0.714–0.898) and 0.757 (95% CI: 0.659–0.855), respectively. In the L3/4 group, the AUC for rPMCSA was 0.754 (95% CI: 0.607–0.900). This indicates that the TFCSA and rPMCSA are powerful diagnostic tools for predicting ULDH. In clinical practice, doctors can predict the risk of ULDH in patients by measuring TFCSA and rPMCSA (22, 23). Thus, personalized and scientific diagnoses and treatment plans can be developed to achieve better treatment outcomes. We further conducted univariate and multivariate logistic regression analysis, and the results showed that both rPMCSA and TFCSA showed a significant correlation with the risk of ULDH (24, 25). The greater the TFCSA, the greater the risk of ULDH occurrence. The larger the rPMCSA, the lower the risk of ULDU occurrence (5).

Paravertebral muscle degeneration is an important disease stage during the natural course of LDH (26–28). The degree of fatty degeneration of the MF in the protruding segment of LDH is significantly higher than that in the contralateral segment, which is a significant reason for chronic LBP in LDH patients (29, 30). This study used the widely recognized VAS to analyze the relationship between the degree of low back pain and MF degeneration in patients with ULDH. The results showed a significant positive correlation between the L1/2 right MF fat area and the L2/3 bilateral MF fat area using the VAS score. This is consistent with the previous view that there is a correlation between chronic low back pain and paravertebral muscle degeneration in patients with lumbar degenerative diseases. Related studies have shown that fat infiltration into the MF increases the likelihood of low back pain by four times (31). The fat content of the MF in patients with chronic nonspecific LBP was significantly higher than that in patients with lumbar spinal stenosis (32). Patients with chronic LBP have significantly reduced paraspinal muscle area and increased fat content, showing a significant correlation between LBP and paraspinal muscle fat infiltration (33, 34). By comparing the symmetry and cross-sectional size of the lumbar MF, we found that patients with low back pain had significantly more atrophy of the lumbar MF than in the asymptomatic healthy volunteers. Changes in muscle area in patients with chronic low back pain are related to their sensitivity to pressure pain (35). This difference may be attributed to the use of different pain measurement methods. The decrease in the MF area is not only related to low back pain, but can also predict the occurrence of short-term low back pain in the future (36). Based on the above findings, we analyzed the relationship between the VAS score and the area of MF fat infiltration and confirmed that the larger the area of MF fat infiltration, the more obvious the LBP in patients with ULDH.

In addition, this study found that the ULDH group had a relatively larger CSA of intervertebral discs and a relatively smaller PM when compared with the healthy control group. Considering that both groups in this study were randomly and consecutively included, we speculated that this result may be related to lumbar disc degeneration. Lumbar disc degeneration is a long-term, chronic, and irreversible process that impairs lumbar stability and disc shock absorption. Research has shown that the height of LIDs gradually decreases with age (37). This may cause the intervertebral disc to protrude towards the surrounding area, resulting in an increase in the corresponding disc area. Lumbar disc degeneration can also lead to compensatory paraspinal muscles, resulting in an imbalanced load and atrophy of the paraspinal muscles. Paravertebral muscle atrophy is highly correlated with the degree of lumbar disc degeneration (38). These findings should be confirmed by expanding the sample size. Previous studies on MF degeneration and LDH mainly focused on the L4/5 and L5/S1 segments (7). LDH degeneration is positively correlated with MF degeneration, and the strengthening training of lumbar muscles is helpful to alleviate lumbar degeneration and muscle atrophy (29). In a study comparing patients with and without nerve root compression to a healthy control group, significant atrophy of the lumbar multifidus muscle was observed at the L4–5 and L5-S1 levels on the affected side in patients with nerve root compression (11). In our study, there was no significant correlation between the protruding direction of LDH and MF degeneration. Studies have shown that there is no relationship between the amount of protrusion and the cross-sectional area of the multifidus muscle (39). In our future research, we should supplement the study on the correlation between ULDH protrusion size and MF degeneration.

Previous studies found that gender may influence the degeneration of paraspinal muscle (40). The atrophy of paraspinal muscle was more severe in female than that in male. In addition, compared with normal people, the degeneration of multifidus muscle was higher in patients with lumbar spinal stenosis under different genders (41). This may be related to muscle content, workload and hormone changes of different genders. Our study show that there was no significant difference in paraspinal muscle parameter between different genders in patients with ULDH. We will expand the sample size for further research in future studies.

Previous studie have found that MF degeneration leads to alterations in the inherent biomechanical relationships of the lumbar spine, increasing the load on the intervertebral discs, which may potentially result in LDH (10). Niu conducted an in-depth exploration of the role of MF in lumbar spine biomechanics through musculoskeletal modeling (42). Incorporating the MF into the musculoskeletal model is significant for enhancing the success rate of simulations and reducing the incidence of overestimation of compressive loads on the lumbar spine. When conducting biomechanical analyses of the lumbar spine, the influence of MF must be taken into account. Studies have revealed that asymmetrical multifidus atrophy has a relatively minor impact on lumbar spine biomechanics. Future research should further investigate the role of MF in lumbar spine biomechanics and explore ways to prevent and treat lumbar degenerative diseases through targeted exercises.

This study has some limitations. Firstly, the sample size was small, therefore calculations in the correlation between intervertebral disc area and ULDH need further research to confirm our results. Secondly, the area measurement method for the paravertebral muscles in this study was manually obtained on MRI axial images, which resulted in certain measurement errors. This study is a single-center observational study geographically confined to Shandong province, China. Variations in ethnicity, environment, and other factors may preclude the extrapolation of our findings to other regions or countries. Future studies, preferably of a multicenter or prospective design, are necessary to corroborate our results across broader populations.

This case-control study analyzed the characteristics of paravertebral muscle degeneration in patients with ULDH and its correlation with chronic nonspecific LBP. MF degeneration was significantly correlated with the occurrence of ULDH. The larger the area of the MF fat infiltration, the more obvious the LBP in patients with ULDH. With the aging of the world population, the incidence rate of lumbar degenerative diseases, such as LDH, is increasing. However, few studies have investigated the correlation between ULDH and paravertebral muscle degeneration, such as in the MF, and clinical studies have often overlooked the impact of MF degeneration on ULDH. Therefore, more in-depth research on the relationship between ULDH and MF degeneration can provide a reference for the prevention, rehabilitation, and treatment of ULDH in clinical practice. Future clinical practices should emphasize the negative impacts of MF degeneration. Patients with ULDH should undergo MR examination to obtain descriptions of MF degeneration. Clinicians should pay attention to MF degeneration when implementing lumbar muscle strengthening training. Lumbar strengthening programs can improve muscle atrophy and lumbar degenerative changes, thereby benefiting the prevention and rehabilitation of ULDH.

## 5 Conclusion

Degeneration of the MF is significantly correlated with the occurrence of ULDH, and the larger the area of fat infiltration in the MF, the more obvious the LBP in patients with ULDH. In addition, TFCSA can serve as an indicator of the occurrence of ULDH.

## Data Availability

The original contributions presented in the study are included in the article/Supplementary Material, further inquiries can be directed to the corresponding authors.
